# Impact of intrahepatic cholestasis of pregnancy on neonatal respiratory outcomes (CHOLE-RESP): protocol for a prospective cohort study

**DOI:** 10.3389/fped.2026.1640579

**Published:** 2026-02-02

**Authors:** Lucia Elena Niculae, Alexandru Stefan Niculae, Raluca Tocariu, Aida Petca

**Affiliations:** 1 Department of Obstetrics and Gynecology, “Carol Davila” University of Medicine and Pharmacy, Bucharest, Romania; 2 Department of Neonatology, “Prof. Dr. Panait Sârbu” Clinical Hospital of Obstetrics and Gynecology, Bucharest, Romania; 3 Department of Pediatric Neurology, “Carol Davila” University of Medicine and Pharmacy, Bucharest, Romania; 4 Department of Obstetrics and Gynecology, Elias University Emergency Hospital, Bucharest, Romania

**Keywords:** biomarkers, intrahepatic cholestasis, pregnancy complications, cohort studies, liver, prospective studies, morbidity, pulmonary surfactants

## Abstract

**Background:**

Intrahepatic cholestasis of pregnancy (ICP) is the most common liver disorder unique to pregnancy, associated with elevated maternal bile acid concentrations and adverse perinatal outcomes.

**Methods:**

This is a prospective cohort study conducted in a level III neonatal unit in Bucharest, Romania. Neonates born to mothers with ICP (serum bile acids ≥10 μmol/L within seven days before delivery) will be enrolled alongside gestational age, sex and birthweight-matched controls. Serum samples will be collected at 24 h, 48–72 h and on day 7 of life. Quantitative ELISA kits will be used to assess serum biomarkers associated with surfactant dysfunction and lung injury. Perinatal and clinical outcomes will be recorded systematically.

**Results:**

The primary aims are to compare the incidence of respiratory distress syndrome (RDS), the need for surfactant administration and serum biomarker profiles between ICP-exposed and unexposed neonates. Secondary aims include evaluating respiratory support requirements, neonatal morbidity and mortality across groups.

**Conclusions:**

We expect that neonates exposed to maternal cholestasis will have a higher incidence of RDS, increased surfactant need, and biomarker alterations consistent with pulmonary compromise. This study may improve understanding of bile acid-related lung injury and inform early risk stratification strategies in affected newborns. (NCT06679972).

## 1 Introduction

Intrahepatic cholestasis of pregnancy (ICP) is the most common hepatobiliary disorder unique to pregnancy, with a reported incidence of 2.9%, depending on geographic and ethnic factors (1). ICP is characterized by maternal pruritus and elevated serum bile acid concentrations, typically emerging during the third trimester. Although maternal symptoms resolve postpartum, ICP is associated with significant perinatal complications, including spontaneous preterm birth, meconium-stained amniotic fluid, fetal distress, and an increased risk of intrauterine demise (2, 3).

Beyond its established association with stillbirth and prematurity, emerging evidence has highlighted a potential link between ICP and neonatal respiratory morbidity. Several studies have demonstrated an increased incidence of RDS among neonates born to mothers with intrahepatic cholestasis of pregnancy, independent of gestational age at delivery (4, 5). Experimental data suggest that elevated fetal bile acid levels may interfere with surfactant synthesis or function, disrupt alveolar integrity and contribute to early pulmonary injury (6). However, the biological pathways underlying this relationship remain poorly understood.

Serum biomarkers of surfactant dysfunction and lung injury have been well characterized in the context of neonatal respiratory complications such as RDS and bronchopulmonary dysplasia (7). Nevertheless, no study to date has systematically evaluated these biomarkers in neonates exposed to maternal cholestasis. Consequently, it remains unknown whether intrauterine exposure to elevated bile acid levels induces biochemical evidence of surfactant impairment or pulmonary injury detectable at birth. Quantifying the extent of lung compromise in this population may not only provide insights into disease pathophysiology, but also support early risk assessment and targeted interventions in the neonatal period.

The CHOLE-RESP study has been designed to address these gaps by prospectively evaluating the incidence of RDS and the need for surfactant therapy in neonates born to mothers with ICP compared to matched controls. In addition, this study seeks to determine whether neonates exposed to intrahepatic cholestasis of pregnancy exhibit distinct serum biomarker profiles consistent with lung injury and surfactant impairment, thereby elucidating a potential link between maternal cholestasis and early neonatal respiratory dysfunction.

## 2 Methods and analysis

### 2.1 Study design

This is a single-center, prospective cohort study conducted in the Neonatology Department of the “Prof. Dr. Panait Sîrbu” Clinical Hospital of Obstetrics and Gynecology in Bucharest, Romania. The study initiated enrolment on October 1st 2024 and is expected to complete recruitment by December 31st 2027, after which follow-up and data analysis will be conducted.

Eligible study participants (neonates born at term or preterm age), enrolled by invitation, will be recruited following the provision of written informed consent by the legal guardian (the mother). The rights and welfare of all participants will be protected in accordance with the principles outlined in the Declaration of Helsinki.

**Figure 1:**
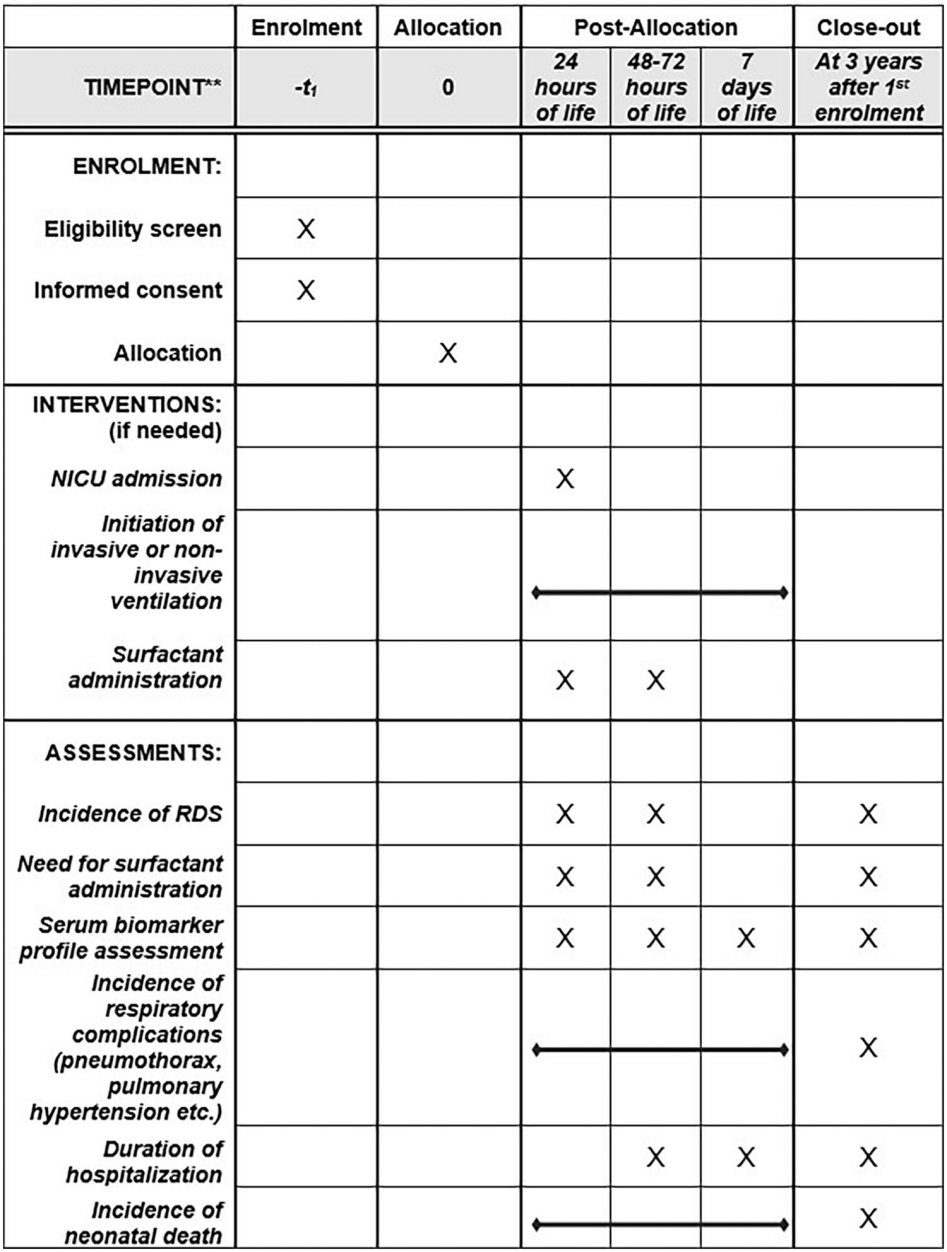
SPIRIT schedule of enrolment, interventions, and assessments. Visual representation of the SPIRIT timeline outlining timepoints for eligibility screening, informed consent, allocation, interventions, and outcome assessments. Samples are scheduled at 24 h, 48–72 h and on day 7 of life (if hospitalized), with follow-up data collected until 3 years after initial enrolment. RDS, respiratory distress syndrome; NICU, neonatal intensive care unit.

**Figure 2:**
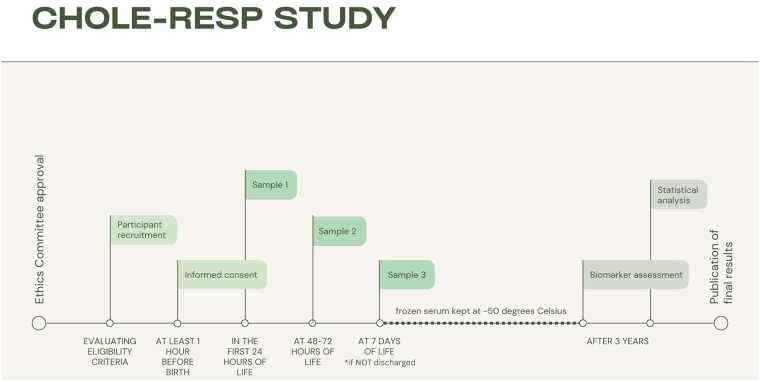
CHOLE-RESP study design timeline. Simplified schematic of the CHOLE-RESP study protocol from ethics approval through sample collection and follow-up.

A SPIRIT schedule and overview of the study design can be found in Figures 1, 2.

This protocol has been developed following the Standard Protocol Items: Recommendations for Interventional Trials checklist (see Supplementary Table 1) (8).

### 2.2 Study objectives

The primary objectives of this study are to evaluate the incidence of RDS, to assess the need for exogenous surfactant administration and to compare serum biomarker profiles indicative of surfactant dysfunction and pulmonary injury between ICP-exposed and unexposed neonates.

For clarity, these are defined as follows:

RDS is defined as a clinical diagnosis made within the first 72 h of life, based on signs of respiratory compromise (tachypnea, grunting, nasal flaring, chest retractions), supportive radiographic findings and a need for supplemental oxygen or respiratory support.

Surfactant administration refers to any administration of exogenous surfactant therapy within the first 72 h of life, regardless of dosing or mode of delivery.

Biomarker profile includes the quantitative assessment of serum biomarkers related to pulmonary injury and surfactant deficiency, measured at 24 h, 48–72 h and on day 7 of life when available.

Secondary objectives include evaluating respiratory morbidity, such as the need for mechanical ventilation and non-invasive respiratory support, as well as the occurrence of serious respiratory complications including pneumothorax, pulmonary hemorrhage and persistent pulmonary hypertension. Additional secondary objectives include assessing neonatal intensive care unit (NICU) admission, length of hospitalization and in-hospital neonatal mortality.

### 2.3 Sample size calculation

An *a priori* power analysis was performed using JASP (version 0.19.2) to estimate the required sample size for detecting a between-group difference in biomarker levels with adequate statistical power. Assuming a one-sided independent samples *t*-test, a medium effect size (Cohen's |*δ*| = 0.5), a Type I error rate (*α*) of 0.05 and a target power (1−*β*) of 0.95, the analysis indicated that a minimum of 66 participants in the ICP-exposed group and 132 in the unexposed group would be required to achieve an actual power of 0.952.

These parameters were selected to align with the study's directional hypothesis that intrahepatic cholestasis of pregnancy is associated with poorer respiratory outcomes and higher biomarker levels. A 2:1 unexposed-to-exposed ratio was adopted to address the lower expected incidence of neonates with maternal ICP, providing greater statistical precision while also enhancing generalizability and minimizing bias, without placing disproportionate demands on recruitment. Also, the higher power threshold of 0.95 was chosen to ensure adequate sensitivity for detecting clinically meaningful biomarker differences, which represent a novel co-primary objective and a central component of the study's design.

This configuration provides robust power to detect moderate effect sizes over 0.5 with high reliability, acknowledging that smaller effects (|*δ*| ≤ 0.25) are associated with a substantially reduced probability of detection. Accordingly, a minimum of 198 neonates will be enrolled to meet the sample size requirements established by the power analysis.

### 2.4 Eligibility criteria

Participants will be enrolled into either the exposed or unexposed group based on predefined eligibility criteria. The exposed group will consist of neonates born to mothers with a recent diagnosis of intrahepatic cholestasis of pregnancy, defined by serum bile acid concentrations exceeding 10 µmol/L within seven days prior to delivery. Furthermore, written informed consent needs to be obtained at least one hour prior to birth. No minimum gestational age at delivery is imposed and eligible neonates must be either born at the study location or transferred to the unit from another neonatal facility within the first 24 h of life. Exclusion from this group will apply in instances where informed consent is not provided, where no serum bile acid measurement is available in the specified time frame or where measured bile acid levels are below 10 µmol/L.

The unexposed group will comprise neonates born at the same institution to mothers without a known history of hepatic disease prior to or during the index pregnancy. Controls will be matched according to gestational age at birth, birthweight and sex. Consistent with the ICP-exposed group, informed consent must be obtained a few hours before delivery. Maternal administration of ursodeoxycholic acid at any point during pregnancy, regardless of dosage or timing, will constitute an exclusion criterion for this group, as it may suggest subclinical hepatic dysfunction.

### 2.5 Sample collection

Venous blood samples (2 mL) will be collected from eligible neonates into gel tubes containing a clot activator, adhering to institutional safety protocols and standard procedures for biological sample handling. Blood collection will be performed within the first 24 h of life, followed by a second sample at 48–72 h, contingent upon clinical stability and the presence of an indication for additional routine laboratory investigations. In neonates with longer hospitalizations, a third sample may be obtained on day 7 of life.

### 2.6 Sample processing

Following blood collection, samples will be immediately transported to the central laboratory for processing. Centrifugation will be performed using an Orto Alresa Microcen 23 centrifuge, operated at 4,000 revolutions per minute (rpm) for 10 min, under room temperature conditions. After centrifugation, the separated serum will be transferred into a single, pre-labeled Eppendorf tube for each participant. All samples will be stored at −50 °C until further analysis. No aliquoting will be performed and all samples will remain under uniform storage conditions throughout the study duration to minimize pre-analytical variability. Upon completion of recruitment, stored serum samples will undergo batch analysis to quantify selected biomarkers associated with surfactant deficiency and pulmonary injury. Quantitative evaluation will be conducted using commercially available sandwich enzyme-linked immunosorbent assay (ELISA) kits validated for use in human serum. To safeguard the novelty of this research, the specific biomarkers evaluated will be disclosed following validation and publication of study findings.

To ensure participant anonymity and maintain blinding during laboratory analyses, each sample will be labeled using a unique alphanumeric code. The coding format will be as follows: “CS” for neonates born to mothers with cholestasis or “FC” for those born to mothers without liver disease, followed by the first initial of the neonate's last name, an optional number for multiple births (1, 2, or 3 for twin or triplet order), a gender identifier (5 for male, 6 for female), and the date of birth in the format DDMM. For example, a male neonate, twin 1, born to a mother with cholestasis, with the last name Niculae, and born on 21st of May, would be assigned the code CSN152105. This code will be recorded on both the primary collection tube and the Eppendorf storage tube. The code list will be securely maintained by the principal investigator, and laboratory personnel responsible for biomarker analysis will remain blinded to the clinical identity and group assignment of each participant.

### 2.7 Data collection and management

Data will be prospectively collected using standardized forms specifically developed for the CHOLE-RESP study. Maternal variables will include demographic characteristics (age and body mass index at delivery), obstetric history, biochemical parameters (serum bile acid concentrations, transaminase levels), and pregnancy-related complications (gestational diabetes mellitus, hypertensive disorders, risk of maternal-fetal infection). Information regarding mode of delivery, use of ursodeoxycholic acid therapy and relevant perinatal events (e.g., meconium-stained amniotic fluid) will also be recorded. Neonatal data will include sex, birthweight, gestational age at delivery, Apgar scores at 1, 5, and 10 min, need for resuscitation, diagnosis of RDS and indicators of respiratory morbidity such as surfactant administration, timing and dosing of surfactant therapy, need for mechanical ventilation or non-invasive respiratory support, oxygen supplementation (FiO₂ requirements), severe respiratory complications including pneumothorax, pulmonary hemorrhage and persistent pulmonary hypertension, NICU admission, duration of hospitalization and neonatal mortality.

Each participant will be assigned a unique anonymized code (as previously described), which will be applied to all clinical records and biological samples. The correspondence between personal identifiers and study codes will be stored separately in a password-protected file accessible exclusively to the principal investigator. Data will be entered into a secure electronic database, with regular cross-verification against source documents to ensure data integrity. Laboratory personnel performing biomarker analyses will remain blinded to group assignment. All data collection and management procedures will comply with institutional ethical guidelines and data protection regulations.

### 2.8 Statistical analysis

Statistical analysis will be performed using JASP 0.19.2. Frequency analysis will be performed using contingency tables. Group differences in the incidence of RDS and the need for surfactant therapy will be assessed using the chi-squared test or Fisher's exact test, where expected cell counts are less than five. Differences in mortality will be evaluated descriptively using odds ratios (ORs), and if event frequency permits, time-to-event analysis may be considered. In addition, time-to-event outcomes such as time to first surfactant dose, time to intubation and time to second surfactant administration will be analyzed using Kaplan–Meier curves and compared using the log-rank test, with Cox regression models applied where appropriate.

Serum biomarker levels indicative of lung injury will be compared between groups at each time point using the Student's *t*-test for normally distributed data or the Mann–Whitney *U* test when normality is not assumed. To assess longitudinal trends in biomarker levels, repeated-measures ANOVA or linear mixed-effects models will be employed, depending on data distribution and completeness. For non-parametric repeated data, the Friedman test may be used. Normality and homogeneity of variances will be tested using the Shapiro–Wilk and Brown-Forsythe tests, respectively. A significance level of 0.05 will be used for all analyses. For normally distributed biomarker data, standardized mean differences between groups will be reported as Cohen's d and Glass's delta. To account for potential residual confounding, gestational age at birth, matched during enrollment, will additionally be included as a covariate in relevant statistical models and stratified analyses may be performed to explore outcome differences across gestational age subgroups.

## 3 Discussion

While intrahepatic cholestasis of pregnancy is widely recognized for its association with preterm birth and fetal complications, a growing body of evidence suggests that neonatal respiratory morbidity may arise independently of gestational age. Clinical case series have proposed that elevated bile acids may directly compromise pulmonary adaptation in affected neonates through mechanisms consistent with bile acid-induced chemical pneumonitis (6). Zecca et al. reported severe respiratory distress in late preterm and early-term infants born to mothers with ICP, despite clinical indicators of lung maturity. The detection of bile acids in bronchoalveolar lavage fluid and the rapid response to surfactant therapy support the hypothesis that bile acid exposure may generate a qualitative surfactant deficit at birth, a pathway that has also been reinforced by animal studies (4, 9–11). More recently, experimental and translational studies have demonstrated that bile acids can impair surfactant synthesis, destabilize the alveolar epithelium and activate inflammatory cascades that hinder postnatal lung adaptation, lending further weight to the concept of bile acid-mediated respiratory injury as a distinct perinatal entity (12–14).

Despite this growing theoretical framework, prospective human data quantifying the extent of pulmonary compromise in neonates exposed to ICP remain scarce. No standardized approach currently exists to assess whether bile acid exposure at clinically relevant concentrations results in measurable surfactant dysfunction or biochemical evidence of lung injury at birth. The CHOLE-RESP study was designed to address this critical knowledge gap. Its prospective design, rigorous matching of controls by gestational age, birthweight, and sex, and integration of both clinical outcomes and serum biomarker analysis represent a structured and comprehensive approach to investigating this emerging hypothesis.

Several design features strengthen the internal validity and feasibility of this study. By including both term and preterm neonates, the study captures the full clinical spectrum of ICP-related deliveries and enables stratified analyses. Also, given the multifactorial nature of neonatal respiratory distress syndrome, statistical models incorporate established clinical risk factors, including gestational age, sex and perinatal infection, to minimize residual confounding and avoid overestimation of the effect of bile acid exposure. Biological samples are collected at defined time points and handled according to standardized protocols, with laboratory personnel blinded to group allocation. The decision to integrate all sampling into clinically indicated procedures minimizes patient risk and reinforces ethical compliance.

Additionally, preliminary retrospective data from our center (June 2020 to December 2024) identified 66 preterm infants born to mothers with ICP among 1,078 preterm births. Although the overall incidence of RDS was not consistently increased, these neonates demonstrated greater respiratory morbidity, with higher requirements for invasive ventilation, non-invasive CPAP and surfactant, as well as longer duration of respiratory support compared with controls. It should be noted that the relatively low number of ICP cases observed retrospectively reflects previous limitations in clinical practice, as bile acid testing was not routinely performed in Romania and often not covered by the public health system. Since October 2024, however, systematic testing for symptomatic mothers and updated obstetric management have been implemented in our institution, already resulting in 54 ICP-exposed neonates recruited within 14 months of prospective enrollment, confirming the feasibility of the study, with control recruitment scheduled to begin after one year. Technical validation of the ELISA kits has been performed in advance and preliminary biomarker analysis will commence once 60 participants are enrolled, with interim results expected to be presented at an international neonatology conference.

Nevertheless, certain limitations are anticipated. As a single-center study, generalizability may be limited, though the use of carefully matched controls mitigates this to some extent. Practical constraints may occasionally lead to delays in sample centrifugation, in which case samples will be stored at 2–5 °C in a dedicated medical-grade refrigerator. Sample hemolysis or insufficient serum volume may affect the ability to test all biomarkers, however these deviations will be documented transparently and addressed in the final analysis. Although early neonatal death is expected to be rare, it may act as a competing risk for outcomes such as RDS. Therefore, all mortality events and their timing will be recorded and considered in the interpretation of respiratory outcomes.

Ethical considerations have been fully integrated into the study design. Informed consent is obtained only after obstetric teams confirm an imminent delivery and before administration of anesthesia. Mothers are given time and autonomy to ask questions and are clearly informed of their right to decline or withdraw participation, without any impact on neonatal care. Blood samples are collected exclusively during procedures already indicated for clinical care, otherwise, if no clinical indication exists or the neonate's condition prohibits collection during a designated window, the sample is skipped and the reason will be included in the study's final reporting.

The CHOLE-RESP study has the potential to significantly advance the field by providing the first structured clinical and biochemical assessment of neonatal respiratory outcomes following ICP exposure. If the study confirms a measurable link between bile acid exposure and pulmonary injury, this could support the development of risk-based screening tools and tailored respiratory management strategies. Moreover, growing evidence from metabolomic and proteomic research suggests that intrahepatic cholestasis of pregnancy is associated with complex molecular signatures extending beyond bile acid concentrations alone (15, 16). Within this framework, the present study lays the groundwork for future multicenter validation efforts, broader biomarker profiling and the integration of hypothesis-generating omics approaches aimed at refining risk stratification and improving outcomes in this understudied neonatal population.

## 4 Ethics and dissemination

The CHOLE-RESP study protocol received approval from the Ethics Committee of the “Prof. Dr. Panait Sîrbu” Clinical Hospital of Obstetrics and Gynecology (Approval No. 34/15.10.2024) and was registered on ClinicalTrials.gov (NCT06679972).

Written informed consent is obtained from the legal guardians of all participants after the obstetric team confirms the likelihood of delivery within the next 24 h and prior to the administration of anesthesia. Mothers are fully informed of the study objectives and procedures, and reassured of their right to decline or withdraw participation at any point without consequences to the medical care provided to their newborn.

Biological samples are collected only during routine clinical blood draws to minimize patient discomfort. In cases where the clinical condition of the neonate precludes timely collection, sampling is deferred to the next scheduled blood test within the collection window. Deviations from the planned sampling protocol, such as refrigeration of unprocessed samples or collection of hemolysed or insufficient serum, are documented and will be transparently addressed in the final manuscript.

Study findings will be disseminated through presentations at scientific conferences and submitted for publication in peer-reviewed journals. Preliminary results will be shared upon completion of the initial recruitment target. No personal identifying information will be published, and all data will be anonymized prior to dissemination.
